# Apparent and standardized ileal amino acid digestibilities of corn, wheat, soybean meal, and corn gluten meal in quail chicks

**DOI:** 10.1016/j.psj.2022.102314

**Published:** 2022-11-04

**Authors:** Mahmoud Ghazaghi, Ahmad Hassanabadi, Mehran Mehri

**Affiliations:** ⁎ Department of Animal Sciences, Faculty of Agriculture, University of Zabol, Sistan, Iran 98661-5538; † Department of Animal Science, Faculty of Agriculture, Ferdowsi University of Mashhad, Mashhad, Iran, 91775–1163

**Keywords:** basal endogenous losses, ileal digestibility, quail

## Abstract

Two experiments were conducted to measure the apparent and standardized ileal digestibilities (AID and SID) of amino acid (**AA**) of corn, wheat, soybean meal (**SBM**), and corn gluten meal (**CGM**) in growing Japanese quail from 14 to 18 (Exp. 1) and 28 to 32 (Exp. 2) d of age. The basal endogenous losses of amino acids were measured by the use of N-free diet. The birds were fed on standard diet before the use of experimental diets. The experimental diets (four ingredients) and N-free diet were randomly assigned to 5 replicate pens (30 birds per pen) and fed for 5 consecutive days. The ileal digesta were collected on d 18 and 32 for the Exp. 1 and Exp. 2, respectively. AID of lysine (Lys) in corn (*P* = 0.047), SBM (*P* < 0.001), and CGM (*P* < 0.001); AID of threonine (Thr) in corn (*P* < 0.001), SBM (*P* < 0.001), and CGM (*P* = 0.075); and AID of isoleucine (Ile) in wheat (*P* < 0.001), SBM (*P* = 0.002), and CGM (*P* < 0.001) were increased as the birds aged. However, AID of methionine (Met) in corn (*P* < 0.001) and CGM (*P* < 0.001), AID of arginine (Arg; *P* < 0.001) and valine (Val; *P* < 0.001) in CGM were lower in younger quails. Among indispensable amino acids, the basal endogenous losses of Thr, Val, and Arg decreased by age (*P* < 0.001). The average of SID of Lys, Ile, Met, Val, Thr, Arg, leucine (Leu), and histidine (His) for corn, wheat, SBM, and CGM were estimated as 83, ND, 89.4, 89.4, 92.1, 90.2, 91.9, and 90.8%; 92.7, ND, 89.1, 93.9, 87.4, 90.2, 89.8, and 88.1%; 90.3, 91.8, 94.3, 90.4, 86.5, 94.0, 84.3, and 95.0%; 82.6, ND, 74.1, 79.6, 84.4, 90.6, 85.2, and 82.4%, respectively. Based on the present study, the AID and SID coefficients of indispensable AA should be adjusted for age classes in Japanese quail during the growing period.

## INTRODUCTION

Two main components of poultry diet are the metabolizable energy and protein source, especially the profile of indispensable amino acid (**IAA**). It has been shown that feed formulation based on apparent ileal amino acid digestibility (**AIAAD**) is more accurate to supply AA requirements than total amino acid (**AA**) basis (Rostagno, et al., 1995). The most important problem of feed formulation based on AIAAD coefficients is the lack of additivity of those coefficients in the mixed diet (Kong and Adeola, 2013; Xue, et al., 2014). On the other hand, Osho, et al. (2019) stated that the standardized ileal amino acid digestibility (**SIAAD**) coefficients of amino acids are more additive than AIAAD coefficients.

As far as the authors are aware, the update data on SIAAD in quail are not available for feed formulation of quail chicks and data derived from broiler chickens are the available basis for feed formulation of growing quail chicks. Therefore, the aim of the present study was to measure the AIAAD of corn, wheat, SBM, and CGM and then to estimate the SIAAD coefficients in quail chicks from 14 to 18 and 28 to 32 d of age.

## MATERIALS AND METHODS

### Bird Management

The Research Animal Ethic Committee of the University of Zabol and Iranian Council of Animal Care approved this experimental protocol. One-day old straight-run quail chicks (Coturnix coturnix Japonica) were provided from the meat-type Quail Genetic Stock Centre at the Research Center of the Research Institute of Zabol (RCRIZ, Sistan, Iran). In Exp 1, a total of 750 quail chicks were randomly allotted to 24 floor pens consisted of 5 treatments with 5 replicates and 30 birds per pen. The chicks were fed on grower diet based on the recommendation of NRC (1994) until d 13. At d 14, the birds received experimental diets for 5 d. At d 18, all birds in pen replicates were killed by Co_2_ asphyxiation and ileal contents were collected. In Exp. 2, the same number of birds in five treatment with 5 replicates and 30 birds per pen were used. The birds received grower diet until d 27. At d 28, the birds were fed on experimental diets for 5 d. At d 32, all birds in pen replicates were killed by Co_2_ asphyxiation and ileal contents were collected. The temperature of experimental house was set at 26°C in the third weeks of age afterward with relative humidity of 60%. The lighting program was 23L:1D during the study. Birds had ad libitum access to feed and water throughout the study.

### Experimental Diets

All feed ingredients were analyzed for CP and AA profile before feed formulation (Table 1). A semipurified N-free diet based on cornstarch was used to measure the basal endogenous losses (**BEL**) of AA and this measurement was then used to estimate SID of AA by correcting AID coefficients for BEL. The remaining 4 treatments in both experiments were formulated based on each ingredient, in which each feed ingredient was the sole source of CP and AA content of the diet (Table 2). Titanium dioxide was added as an indigestible marker at 5 g/kg of diet. All diets were fed in mash form.

**Table 1:** Concentration of amino acids in test ingredients (% as is basis).

	Corn	Wheat	Soybean meal	Corn gluten meal
Dry matter	88.1	92.7	92.3	95.1
Crude protein	8.80	11.9	46.9	61.0
Methionine	0.177	0.186	0.641	1.393
Lysine	0.244	0.351	2.872	0.928
Threonine	0.299	0.336	1.862	1.911
Tryptophan	0.063	0.140	0.636	0.323
Valine	0.395	0.501	2.292	2.630
Arginine	0.402	0.581	3.375	1.812
Isoleucine	0.282	0.395	2.212	2.298
Leucine	0.987	0.776	3.655	9.255
Histidine	0.243	0.278	1.244	1.165
Glutamic acid	1.348	3.500	8.785	12.657
Aspartic acid	0.538	0.609	5.462	3.480
Serine	0.400	0.534	2.404	2.833
Glycine	0.330	0.474	2.049	1.579
Alanine	0.614	0.424	2.092	4.988
Proline	0.744	0.140	2.430	5.152

**Table 2:** Composition of experimental diets.

Ingredient (%)	N-free diet	Corn gluten meal	Corn	Soybean meal	Wheat
Test ingredient	-	30.95	80.0	40.0	88.0
Corn starch	66.26	64.14	16.33	55.38	7.30
Sucrose	24.30	-	-	-	-
Solka-floc	5.00	-	-	-	-
Dicalcium Phosphate	1.50	0.84	-	1.27	1.39
K_2_CO_3_	1.36	0.50	0.36	-	-
NaHCO_3_	-	0.50	0.50	-	-
TiO_2_	0.50	0.50	0.50	0.50	0.50
Vitamin premix^1^	0.25	0.25	0.25	0.25	0.25
Mineral premix^2^	0.25	0.25	0.25	0.25	0.25
NaCl	0.38	0.36	0.20	0.35	0.31
MgO	0.10	0.10	0.10	1.00	1.00
Choline chloride	0.10	0.10	0.10	1.00	1.00
CaCO3	-	1.51	1.41	-	-
Calculated composition					
AME (kcal/kg)	3410	3590	3400	3620	2830
Crude protein (%)	-	18.0	6.34	19.50	12.32
Ca (%)	0.80	1.00	1.00	1.00	1.00
Na (%)	0.15	0.16	0.23	0.15	0.16
K (%)	0.60	0.40	0.40	0.80	0.40
Cl (%)	0.23	0.23	0.15	0.23	0.23
DEB^3^ (mEq/kg)	210.0	311.9	118.6	206.1	242.1
Crude fiber (%)	4.95	2.40	1.76	2.80	2.64
NSP^4^ (%)	-	2.34	7.55	12.30	10.60

1 Mineral premix provided per kilogram of diet: Mn, 100 mg; Se, 0.2 mg; I, 1.0 mg; Cu, 100mg; Fe, 50 mg.

2 Vitamin premix provided per kilogram of diet: vitamin A, 11,000 IU; cholecalciferol, 2,300 IU; vitamin E, 121 IU; vitamin K_3_, 2.0 mg; vitamin B_12_, 0.02 mg; thiamin, 4.0 mg; riboflavin, 4.0 mg; folic acid, 1.0 mg; biotin, 0.03 mg; pyridoxine, 4.0 mg; choline chloride, 840 mg; ethoxyquin, 0.125 mg.

3 DEB: Dietary electrolyte balance.

4 NSP: Non starch polysaccharides.

### Digesta Sampling

The ileal digesta (between Meckel's diverticulum to approximately 1cm proximal to the ileocecal junction) were collected by gently flushing with distilled water into plastic containers and samples within a pen were pooled, frozen, and stored at −20°C until further processing. Samples were freeze-dried, ground by the use of a mortar and pestle, and submitted to the laboratory for amino acid and titanium analysis.

### Chemical Analysis

As described by Hasanvand et al. (2018), feed samples were prepared using a 24-h hydrolysis in 6 N hydrochloric acid at 110°C under an atmosphere of nitrogen. For Met and Cys, performic acid oxidation was done before acid hydrolysis. Samples for Trp analysis were hydrolyzed using barium hydroxide. Chromatographic separations of amino acids were performed with a Waters HPLC system (Waters, Milford, MA). It consisted of a 1525 Binary HPLC pump, a 2,487 Dual λ absorbance detector operating at 254 nm, Breeze chromatography software and a Rheodyne 7725 injection valve (Cotati, CA) which equipped with a 20 μL sample loop. The column was Pico tag (3.9 × 150 mm I.D.; particle size 5 μm). Titanium dioxide was determined by hydrolysis of the sample with sulphuric acid (H_2_SO_4_) followed by a color reaction as described by Short et al. (1996).

### Calculations

The BEL, AID, and SID coefficients of AA were calculated according to Adedokun et al. (2008):

$$\text{BEL}{\;}_{\left(mg/kgDMI\right)}=\text{amino}\;\text{acid}\;\text{in}\;\text{ileal}\;\text{digest}a_{\left(\text{mg}/\text{kg}\right)}\times \frac{diet\;titanium\;\left(mg/kg\right)}{ileal\;titanium\;\left(mg/kg\right)}$$

$$AIAAD_{\%}=\left[1-\left(\frac{titanium\;in\;diet}{titanium\;in\;ileal\;digesta}\right)\times \left(\frac{amino\;acid\;in\;digesta}{amino\;acid\;in\;diet}\right)\right]$$

$$SIAAD_{\%}=AIAAD_{\%}+\left[\frac{BEL_{\left(mg/kgDMI\right)}}{amino\;acid\;content\;of\;raw\;material_{\left(mg/kgDM\right)}}\right]\times 100$$

### Statistical Analysis

Data were analyzed by using PROC GLM of SAS (2002) as a completely randomized design. Effect of age on BEL, AIAAD and SIAAD values was determined. The level of significance was set at *P* < 0.05.

## RESULTS

No mortality was observed during the study and the quail performance was shown in Table 3. In Exp. 1, the highest performance was observed in the birds fed on wheat (*P* < 0.001). In Exp. 2, the birds fed on SBM had the highest gain and gain:feed compared to the other groups (*P* < 0.001). In both experiments, the birds fed on N-free diet had the lowest performance compared to the other groups (*P* < 0.001). Digestibility coefficients were feed and age-dependent. AIAAD and SIAAD coefficients of AA for corn, wheat, SBM, and CGM at 2 different classes' age were shown in Table 4, Table 5, Table 6, Table 7.

**Table 3:** Quail performance during 14 to 18 (I) and 28 to 32 d (II) of age (n = 150).

Ingredient	I			II		
	Gain (g/b)	Feed intake (g/b)	Gain:feed (g/g)	Gain (g/b)	Feed intake (g/b)	Gain:feed (g/g)
Corn	−12.3	33.1	−0.376	−10.3	70.0	−0.143
Wheat	8.30	49.0	0.168	6.06	89.0	0.066
Soybean meal	5.74	43.3	0.131	6.45	168	0.039
Corn gluten meal	−4.42	35.3	−0.126	−7.96	67.4	−0.115
N-free diet	−20.1	30.7	−0.655	−32.3	41.4	−0.782
SEM	2.00	1.20	0.06	4.66	8.79	0.08
*P*-value	< 0.001	< 0.001	< 0.001	< 0.001	< 0.001	< 0.001

**Table 4:** Estimated marginal means of apparent and standardized ileal amino acid digestibility (AIAAD and SIAAD) of corn in quail chicks at two different age classes (n = 150).

Amino acid (AA)	AIAAD (%)		SIAAD (%)	
	I	II	I	II
Indispensable				
Lysine	70.2	79.7	84.0	82.0
Isoleucine	75.6	79.3	92.2	ND
Methionine	92.4	81.5	94.5	84.2
Valine	84.8	84.6	93.9	85.7
Threonine	79.8	95.4	88.3	95.8
Arginine	87.0	89.8	89.8	90.6
Histidine	94.3	83.3	97.5	84.0
Dispensable				
Glutamic acid	89.2	79.3	90.2	80.2
Aspartic acid	71.4	94.1	74.3	94.6
Leucine	93.6	88.9	94.1	89.8
Serine	84.6	83.3	86.9	84.0
Glycine	91.2	70.4	92.1	70.5
Alanine	90.5	82.4	91.1	85.3
Proline	95.9	63.1	96.2	66.3
Pooled SEM	1.50	1.06	1.57	1.11
Probability				
AA	< 0.001		< 0.001	
Age	< 0.001		< 0.001	
AA × Age	< 0.001		< 0.001	

I: 14 to 18 d of age; II: 28 to 32 d of age.

ND: not determined.

**Table 5:** Estimated marginal means apparent and standardized ileal amino acid digestibility (AIAAD and SIAAD) of wheat in quail chicks at two different age classes (n = 150).

Amino acid (AA)	AIAAD (%)		SIAAD (%)	
	I	II	I	II
Indispensable				
Lysine	86.5	88.5	95.5	90.0
Isoleucine	79.5	85.7	ND	ND
Methionine	90.8	83.8	92.5	94.5
Valine	88.3	85.0	94.7	93.0
Threonine	82.6	81.5	89.8	92.9
Arginine	84.3	76.7	88.5	92.0
Histidine	81.2	88.8	91.2	92.1
Dispensable				
Glutamic acid	74.3	73.9	86.7	90.2
Aspartic acid	71.4	92.9	84.0	95.4
Leucine	78.0	92.9	84.2	95.4
Serine	89.3	90.0	90.8	94.2
Glycine	84.1	88.8	92.1	94.1
Alanine	93.6	76.4	95.2	93.3
Proline	93.5	70.0	94.3	89.6
Pooled SEM	2.12	1.50	2.15	1.52
Probability				
AA	< 0.001		< 0.001	
Age	< 0.001		< 0.001	
AA × Age	< 0.001		0.005	

I: 14 to 18 d of age; II: 28 to 32 d of age.

ND: not determined.

**Table 6:** Estimated marginal means of apparent and standardized ileal amino acid digestibility (AIAAD and SIAAD) of soybean meal in quail chicks at two different age classes (n = 150).

Amino acid (AA)	AIAAD (%)		SIAAD (%)	
	I	II	I	II
Indispensable				
Lysine	82.0	97.5	83.1	97.6
Isoleucine	85.3	95.5	87.8	95.7
Methionine	94.7	92.7	95.3	93.2
Valine	81.5	97.4	83.1	97.6
Threonine	80.1	91.5	81.5	92.8
Arginine	91.1	96.5	91.5	96.6
Histidine	95.2	93.9	95.9	94.1
Dispensable				
Glutamic acid	90.5	87.8	90.6	88.5
Leucine	80.5	86.6	81.9	86.7
Serine	89.4	94.5	89.7	94.6
Glycine	93.3	95.0	93.5	95.2
Alanine	86.8	82.1	87.0	82.9
Proline	95.3	87.6	95.4	88.4
Pooled SEM	1.79	1.27	1.76	1.25
Probability				
AA	< 0.001		< 0.001	
Age	< 0.001		< 0.001	
AA × Age	< 0.001		< 0.001	

I: 14 to 18 d of age; II: 28 to 32 d of age;

ND: not determined.

**Table 7:** Estimated marginal means of apparent and standardized ileal amino acid digestibility (AIAAD and SIAAD) of corn gluten meal in quail chicks at two different age classes (n = 150).

Amino acid (AA)	AIAAD (%)		SIAAD (%)	
	I	II	I	II
Indispensable				
Lysine	67.8	93.8	71.2	93.9
Isoleucine	63.1	65.2	ND	ND
Methionine	86.0	61.6	86.3	61.9
Valine	83.7	74.0	85.1	74.2
Threonine	76.8	90.6	78.0	90.7
Arginine	89.5	90.9	90.1	91.0
Histidine	67.5	90.3	74.4	90.5
Dispensable				
Glutamic acid	86.8	88.6	86.9	89.1
Leucine	76.2	93.6	78.2	94.1
Serine	94.4	71.8	94.7	73.8
Glycine	78.2	93.0	78.5	93.8
Alanine	83.1	89.7	84.1	90.1
Proline	85.7	93.1	87.7	93.4
Pooled SEM	3.11	2.20	3.27	2.32
Probability				
AA	< 0.001		< 0.001	
Age	< 0.001		< 0.001	
AA × Age	0.035		< 0.001	

I: 14 to 18 d of age; II: 28 to 32 d of age; ND: not determined.

**Figure 1:**
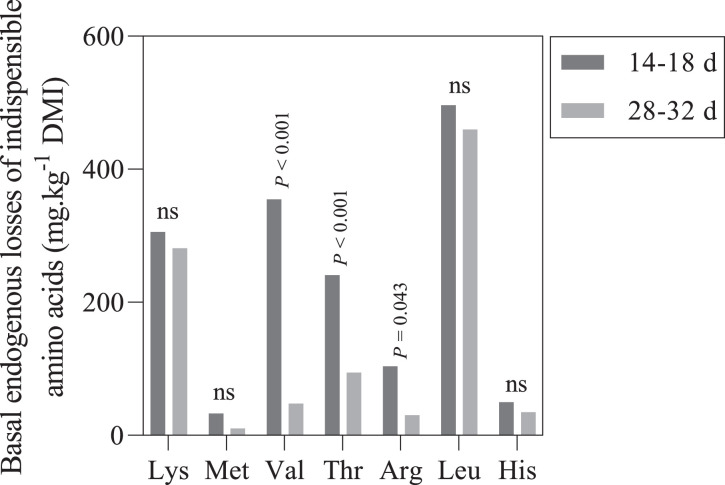
Basal endogenous losses of some indispensable amino acids (e.g., Lys: lysine; Met: methionine; Val: valine; Thr: threonine; Arg: arginine; Leu: leucine; His: histidine) at two different age classes.

As shown in Figure 1, basal endogenous losses of Thr (*P* < 0.001), Val (*P* < 0.001), and Arg (*P* = 0.043) were decreased by age, while the decreasing changes of the remaining indispensable AA were not significant.

**Figure 2:**
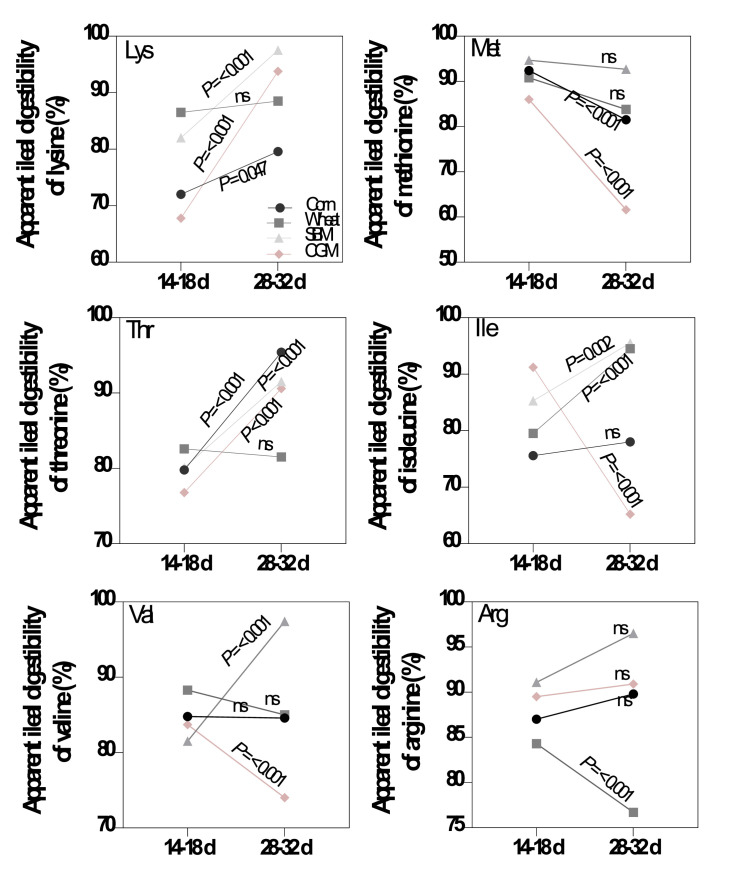
Sharp changes in apparent ileal digestibility of lysine, threonine, valine, isoleucine, and arginine of corn, wheat, soybean meal (SBM), and corn gluten meal (CGM) with increasing age in quail chicks.

For corn, at wk 3, the lowest and highest SIAAD coefficients were determined for aspartic acid (**Asp**) and His, respectively. At wk 5, the corresponding values were determined for glycine (**Gly**) and Thr, respectively. The AID of Lys (*P* = 0.047) and Thr (*P* < 0.001) were increased by age while the AID of Met (*P* < 0.001) was decreased with increasing age (Figure 2). The average of AID and SID of the studied AA were shown in Table 8.

**Table 8:** The average of apparent and standardized ileal amino acid digestibility (AIAAD and SIAAD; %) of corn, wheat, soybean meal (SBM), and corn gluten meal (CGM) in quail chicks.

Amino acid	Corn		Wheat		SBM		CGM	
	AIAAD	SIAAD	AIAAD	SIAAD	AIAAD	SIAAD	AIAAD	SIAAD
Indispensable								
Lysine	75.8	83.0	87.5	92.7	89.7	90.3	80.8	82.6
Isoleucine	77.4	ND	87.0	ND	90.4	91.8	78.2	ND
Methionine	87.0	89.4	87.3	89.1	93.7	94.3	73.8	74.1
Valine	84.7	89.8	90.6	93.9	89.5	90.4	78.9	79.6
Threonine	87.6	92.1	82.1	87.4	85.8	86.5	83.7	84.4
Arginine	88.4	90.2	88.5	90.2	93.8	94.0	90.2	90.6
Leucine	91.2	91.9	85.4	89.8	83.5	84.3	84.9	85.2
Histidine	88.8	90.8	85.0	88.1	94.6	95.0	78.9	82.4
Dispensable								
Glutamic acid	84.3	85.2	78.9	80.5	89.1	89.6	87.7	88.0
Aspartic acid	82.7	84.4	82.1	84.9	ND	ND	ND	ND
Serine	84.0	85.5	91.7	92.5	91.9	92.2	83.1	83.2
Glycine	80.8	83.1	90.0	91.1	94.1	94.2	85.6	85.8
Alanine	86.4	88.2	91.9	94.2	84.5	85.0	86.4	86.6
Proline	79.5	81.2	81.8	85.0	91.5	91.9	89.4	89.6
SEM	1.01	0.96	1.30	0.89	1.10	1.08	1.91	2.01

ND: not determined.

For wheat, at wk 3, the lowest and highest SIAAD coefficients were determined for Asp and Lys, respectively. At wk 5, the corresponding values were determined for Pro and Leu + Asp, respectively. AID of Ile (*P* < 0.001) increased with increasing age while AID of Arg (*P* < 0.001) decreased with increasing age (Figure 2).

For SBM, at wk 3, the lowest and highest SIAAD coefficients were determined for Thr and His, respectively. At wk 5, the corresponding values were determined for Ala and Lys + Val, respectively. AID of Lys (*P* < 0.001), Thr (*P* < 0.001), Ile (*P* = 0.002), and Val (*P* < 0.001) increased with increasing age (Figure 2).

For CGM, at wk 3, the lowest and highest SIAAD coefficients were determined for Lys and Ser, respectively. At wk 5, the corresponding values were determined for Met and Leu, respectively. AID of Lys (*P* < 0.001) and Thr (*P* < 0.001) increased with increasing age while AID of Ile (*P* < 0.001), Met (*P* < 0.001), and Val (*P* < 0.001) decreased with increasing age (Figure 2).

## DISCUSSION

Because of scanty data on AA digestibility in quail, we have to compare our data with that reported Garcia et al. (2007) and Kim et al. (2012) by on broiler and rooster.

**Figure 3:**
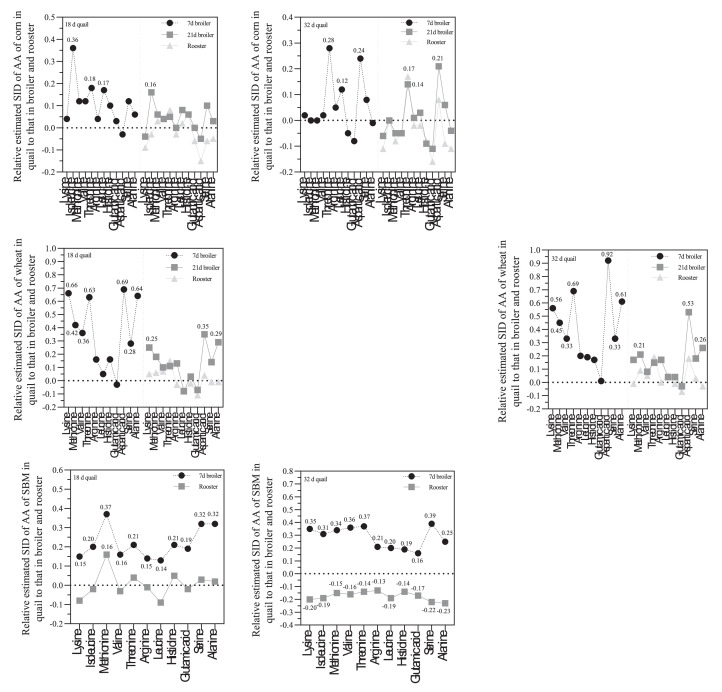
Relative standardized ileal digestibility (SID) of amino acids (AA) of corn, wheat, and soybean meal in quail to those in broiler and rooster as reported by Garcia et al. (2007).

For corn, the comparison our data with Garcia et al. (2007) showed that SID coefficients of almost all tested AA in 18 d quail were higher than those reported for 7-day-old broilers. The SID of Ile, Thr, and leu were 36, 18, and 17% higher than those reported for 7-day-old broilers. The SID of Thr, Leu, and Asp in 32-day-old quail were 28, 12, and 24% higher than those reported for 7-day-old broilers (Figure 3). It was clearly that growing quail was more efficient than broiler and rooster to digest tested AA, which was in agreement with Vasan et al. (2008) who showed that almost all indispensable AA of corn in quail had higher digestibility values than rooster. As shown in Figure 3, the AA digestibilities in 32-day-old quail were comparable with adult rooster.

The differences between AA digestibility values of wheat in quail chicks and broilers were very higher than those reported for corn. The SID of Lys, Met, Val, Thr, Asp, Ser, and Ala in 18-day-old quail were 66, 42, 36, 63, 69, 28, and 64% higher than those in 7 d-old broilers and the corresponding values in 32-day-old quail were 56, 45, 33, 69, 92, 33, and 61% higher than those in 7 d-old broilers. The SID of Met, Asp, and Ala in 32-day-old quail were 21, 53, and 26% higher than 21-day-old broilers. These differences between quail and 21-day-old broilers and rooster were relatively lower than those between younger quails and broilers (Figure 3). Although Garcia et al. (2007) reported that the digestibility coefficients for all AA in tested ingredients were significantly lower at 7-day-old broilers than adult roosters, the comparison our data showed that young quails had higher efficiency to digest AA than roosters.

The comparison our data for SBM with that reported by Garcia et al. (2007) revealed that 18-day-old quail had higher digestibilities for all tested AA than 7-day-old broilers, in which the SID of Lys, Ile, Met, Val, Thr, Arg, Leu, His, Glu, Ser, and Ala in 18-day-old quail were 15, 20, 37, 16, 21, 15, 14, 21, 19, 32, and 32% higher than 7-day-old broilers. Digestibility values in 18-day-old quail and roosters were comparable. In 32-day-old quail, the corresponding values were 35, 31, 34, 36, 37, 21, 20, 19, 16, 39, and 25% higher than those in 7-day-old broilers. However, the SID of the all AA in 32-day-old quail was lower than those in roosters (Figure 3).

**Figure 4:**
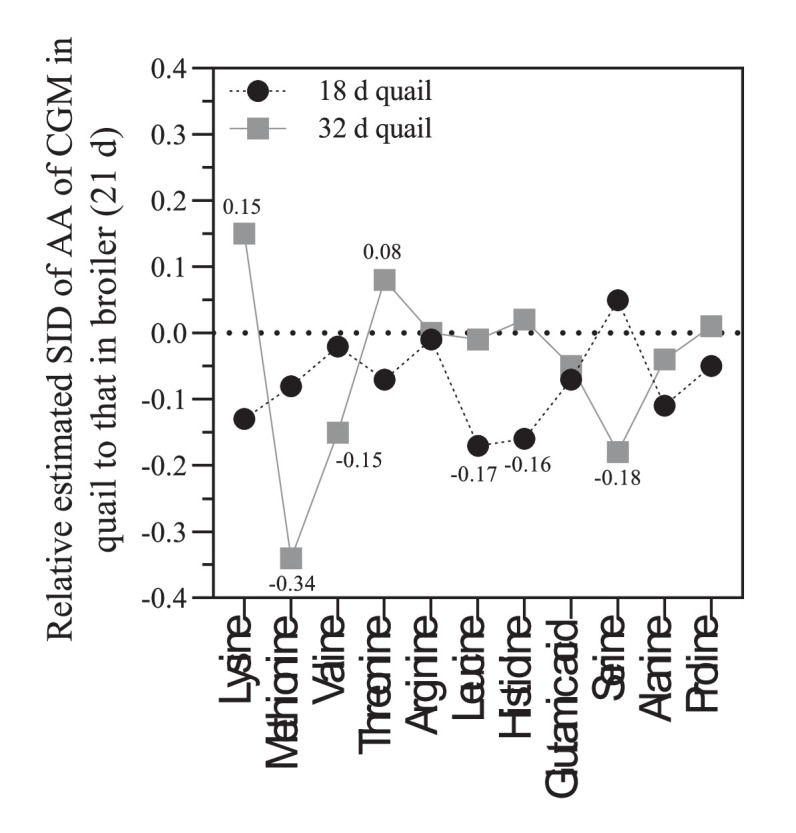
Relative standardized ileal digestibility (SID) of amino acids (AA) of corn gluten meal (CGM) in quail (14–18 and 28–35 d) to those in broiler (21 d) as reported by Kim et al. (2012).

The SIAAD coefficients of CGM in quail were compared with data of 21-day-old broiler reported by Kim, et al. (2012). Digestibilities of almost all tested AA in 18-day-old quail were lower than those in 21-day-old broilers (Figure 4). The SID of tested AA in 32-day-old quail was higher or comparable with 21-day-old broilers with exception of Met and Ser. The SID of Lys and Thr in 32-day-old quail were 15 and 8% higher than broilers.

The present data revealed that digestibility values of AA were dependent on feed ingredient and specific AA, quail chick had a higher ability for digestion and digestions of AA than broilers, particularly for corn, wheat, and CGM. More studies are warranted in the field of feed assessment for quail, especially in terms of AA digestibilities.

## DISCLOSURES

The authors whose names are listed immediately below certify that they have NO affiliations with or involvement in any organization or entity with any financial interest (such as honoraria; educational grants; participation in speakers’ bureaus; membership, employment, consultancies, stock ownership, or other equity interest; and expert testimony or patent-licensing arrangements), or nonfinancial interest (such as personal or professional relationships, affiliations, knowledge, or beliefs) in the subject matter or materials discussed in this manuscript.
